# Distribution characteristics of trace mineral elements in tea garden soils of Guizhou region and their biological accumulation and response to tea quality

**DOI:** 10.7717/peerj.21210

**Published:** 2026-05-20

**Authors:** Wange He, Zuyong Chen, Shan Xie, Yuansheng Liu, Yingge Shu

**Affiliations:** College of Agronomy, Guizhou University, Guiyang, Guizhou, China

**Keywords:** Tea quality, Biological accumulation, Trace mineral elements, Guizhou tea garden soils

## Abstract

**Background:**

*Camellia sinensis*, a vital cash crop in southwest China, makes Guizhou Province renowned for its premium zinc-selenium (Zn-Se)-enriched tea. While soil trace elements influence tea plant growth and quality, previous studies have predominantly focused on geochemical features, leaving gaps in understanding their distribution in tea plantations, transfer to tea plants, and specific impacts on tea quality. This study investigates trace element distribution in the rock-soil-tea system across Guizhou’s major tea-growing regions, assesses their effects on tea quality, and identifies key factors regulating soil element levels, aiming to underpin the expansion of high-quality Zn-Se tea production and sustainable regional economic development.

**Methods:**

Drawing on a prior survey of 146 surface soil (0 ∼ 20 cm) samples from Guizhou tea gardens, 36 representative tea gardens spanning 12 local tea-producing areas were chosen for field research. Soil profile samples (0 ∼ 20 cm, 20 ∼ 40 cm, 40 ∼ 60 cm) and tea leaves were collected to analyze the spatial variability of major minerals (aluminum (Al), iron (Fe), calcium (Ca), magnesium (Mg), phosphorus (P), potassium (K), sulfur (S)) and trace elements (manganese (Mn), copper (Cu), zinc (Zn), molybdenum (Mo), germanium (Ge), selenium (Se), strontium (Sr)), as well as trace element bio-accumulation and their correlations with tea quality. Soil and rock samples were digested with aqua regia, tea samples with concentrated HNO_3_, and elemental concentrations were quantified using inductively coupled plasma mass spectrometry (ICP-MS).

**Results:**

Results revealed significant spatial heterogeneity in soil trace elements (Mn, Cu, Zn, Mo, Ge, Se, Sr), with Cu and Se showing the highest variability, followed by Zn and Sr, then Mn and Mo, while Ge exhibited the least variation. In surface soils, Se concentrations ranged from 0.23 to 1.50 mg kg^−1^ and Zn from 33.0 to 105.6 mg kg^−1^; 69.4% of surface soils met Se-enriched standards, but only 33.3% reached Zn-Se-enriched levels. Strong positive correlations were detected between soil and parent rock contents of Mn, Cu, Zn, Mo, Se, and Sr, particularly for Se and Cu. Geological age and parent rock type were identified as dominant factors shaping soil Zn and Se levels, with synergistic interactions among elements further promoting their enrichment in soils. Trace element levels in tea leaves varied markedly across regions (*P* < 0.01), with Se showing the highest variability, followed by Zn and Sr. Biological accumulation coefficients ranked as Mn > Zn > Cu > Se > Sr > Ge > Mo. Zn and Se contents in leaves were strongly correlated with corresponding soil concentrations (*P* < 0.01), especially for Se. Leaf Se ranged from 0.05 to 0.54 mg kg^−1^, and Zn from 24.4 to 97.2 mg kg^−1^. Among samples, 25.0% met the threshold for Se-enriched tea and 22.2% for Zn–Se co-enriched tea, with the latter showing clear geographic clustering consistent with soil Zn and Se distribution.

## Introduction

Tea is one of the world’s three most widely consumed beverages. Zinc-selenium (Zn-Se)-enriched tea contains active zinc and organic selenium, while Ge-enriched tea contains tea polyphenols, germanium, and organic germanium. These components offer significant nutritional value and health benefits, thereby increasingly attracting consumer interest. Tea cultivation is a crucial ecological industry in Guizhou Province. By 2024, the total tea plantation area had reached 467,000 hectares, with tea gardens distributed across all nine prefecture-level administrative divisions in the province. Currently, Guizhou has established a regionally distinct tea industry specializing in the production of high-quality and Zn-Se-enriched teas (The People’s Government of Guizhou Province, 2024). The geological environment and soil conditions are key factors influencing tea quality (Han, Bi & Yang, 2010; Ren, Zhao & Chen, 2012; Zhou et al., 2015; Yu et al., 2020; Yang et al., 2025; Shu et al., 2025). *Camellia sinensis* absorbs various nutrients from the soil through its roots, including essential mineral elements such as calcium (Ca), magnesium (Mg), iron (Fe), manganese (Mn), copper (Cu), Zn, and Se. These elements play indispensable roles in the growth and metabolic processes of tea plants. Particularly, trace elements such as Zn, Se, and germanium (Ge) in tea garden soils not only influence the normal growth of *Camellia sinensis* but also significantly affect tea quality (Zhang et al., 2007; Liu et al., 2010; Duan & Chen, 2018; Dong et al., 2022; Su, An & Yu, 2024). *Camellia sinensis* is an economically important crop with a strong capacity for selenium enrichment. It can convert non-biologically active inorganic selenium into safe and effective organic selenium through biological enrichment and transformation processes. Tea leaves are the primary site of selenium accumulation, and the resulting Se-enriched tea can serve as an ideal dietary supplement for human selenium intake (Ai et al., 2019; Zhang et al., 2020a; Zhang et al., 2020b; Dong et al., 2022), such as Fenggang Zn-Se-enriched tea and Kaiyang Se-enriched tea. However, the presence of Zn-Se-enriched tea is closely related to the levels of Zn and Se in the soil of the tea-growing regions. According to the Inspection and Research Report on Naturally Se-enriched Tea in Guizhou Province, analyses of tea samples from 40 tea farms across 21 counties and cities in Guizhou revealed that the Se content in tea leaves ranged from 0.40 mg kg^−1^ to 4.11 mg kg^−1^, with an average of 1.08 mg kg^−1^. The main selenium-rich tea-producing areas are concentrated in Fenggang County, Meitan County, Kaiyang County, Duyun City, and Puan County. Another study (Liu, 2009) reported that the average Ge content in the soils of Yanyin County, Guizhou, reached 2.17 mg kg^−1^, meeting the criteria for Ge-enriched soil. In China, extensive research has been conducted on Zn-Se-enriched tea, leading to the formulation of national and local standards such as “Soil selenium grade” National Administration for Market Regulation of the People’s Republic of China (2024) _GB/T 44971-2024 and “Product of geographical indication Fenggang Zinc-selenium Tea” Guizhou Provincial Market Regulatory Administration (2015) _DB52/T 489-2015. However, research on Ge-enriched tea remains relatively limited, with only a few studies addressing Ge content in tea (Yang, Xiao & Miao, 2020; Su, An & Yu, 2024); Additionally, there are currently no reports on strontium (Sr) content in tea garden soils or its bioaccumulation characteristics. Based on recent research data, more studies have focused on the geochemical characteristics of Zn, Se, Ge, and Sr in agricultural soils (Ye et al., 2015; Liu et al., 2015; Zhang et al., 2018; You, Bao & Li, 2020; Han et al., 2020; Zhang et al., 2020a; Zhang et al., 2020b; Wei, Guo & Qiao, 2021; Wang & Yuan, 2023; Chen, 2024). Variations in regional rock types and soil mineral composition are considered key factors influencing the levels of Zn, Se, Ge, and Sr in tea (Wang et al., 2017; Song, Pan & He, 2018; Han et al., 2018; Ma & Lin, 2019; Yu et al., 2020; Zhang et al., 2020a; Zhang et al., 2020b; Sun et al., 2025).

Numerous studies have been conducted on the migration and accumulation mechanisms of Zn and Se between soil and tea leaves (Duan & Chen, 2018; Peng et al., 2021; Dong et al., 2022). A significant correlation has been observed between the concentrations of Zn and Se in garden soils and those in tea leaves. However, systematic research on the migration and accumulation mechanisms of Ge and Sr between soil and tea leaves remains limited (Hu et al., 2019; Liu et al., 2022; Su, An & Yu, 2024; Sun et al., 2024). From a biogeochemical perspective, it is evident that understanding the content and spatial distribution patterns of mineral elements within the rock-soil-tea system in Guizhou’s major tea-producing areas-particularly the distribution characteristics of trace mineral elements in tea garden soils and their relationship with tea quality is crucial for the establishment of large-scale, industrialized production bases for health-promoting teas, such as Zn-Se-enriched tea. Therefore, based on the previous survey of soils in tea gardens in Guizhou region and research results (Yang et al., 2023), this study investigates the distribution characteristics of trace elements in rocks and soils across different tea-producing areas and their influence on tea quality through field investigations and soil sampling in Guizhou’s main tea-growing regions. The findings aim to provide a scientific foundation for the large-scale development of high-quality and Zn-Se-enriched tea, thereby promoting the sustainable economic development of the region.

## Materials and Methods

### Regional study overview

**Figure 1:**
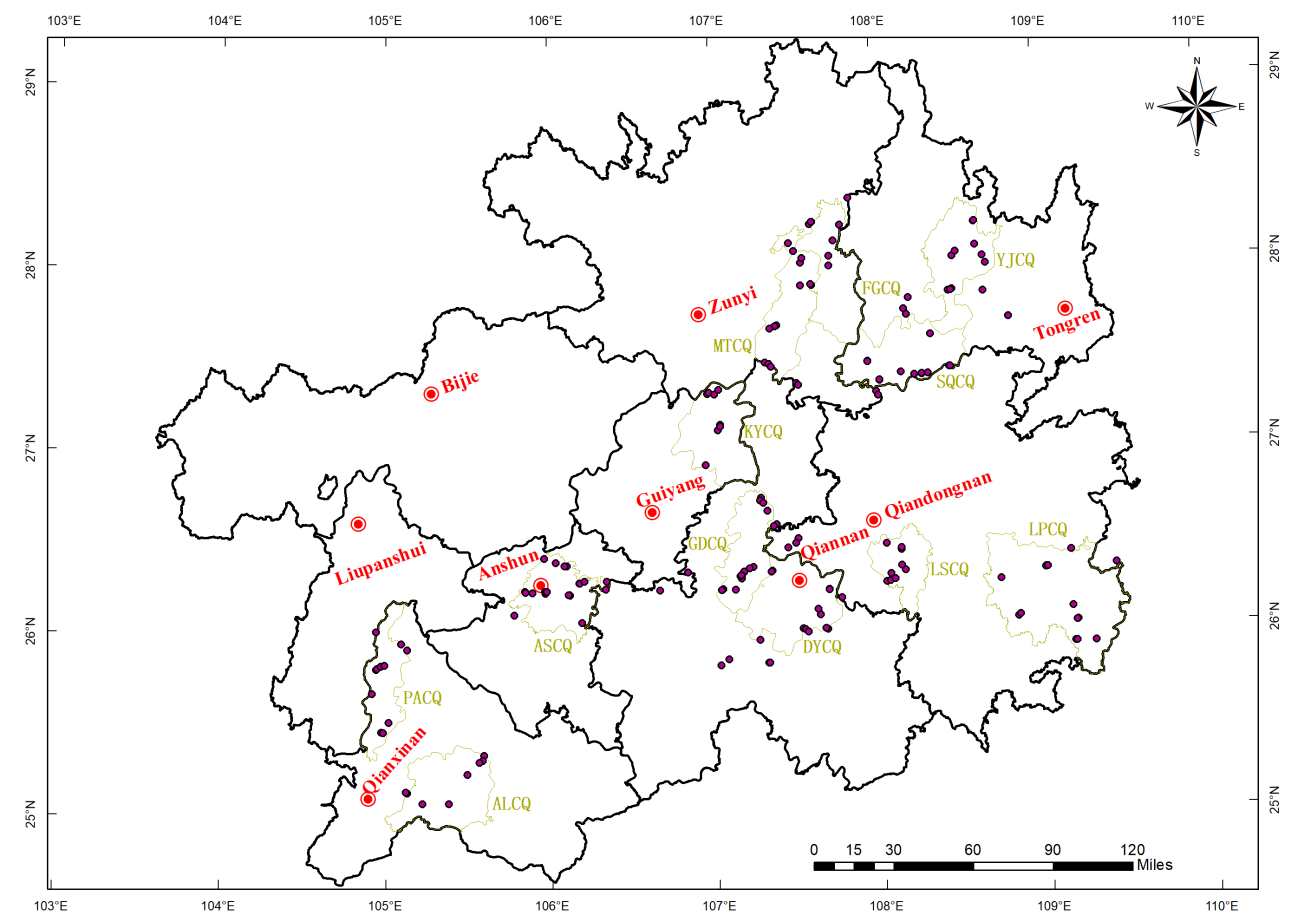
Schematic diagram of the geographical location of the main tea-producing areas in Guizhou region.

Guizhou Province is geographically situated between 103°36′E and 109°35′E as well as 24°37′N and 29°13′N, in the eastern segment of the Yunnan-Guizhou Plateau of China. It falls under a subtropical humid monsoon climate, characterized by an average annual temperature of 15.3 °C and an annual precipitation of 1,100 ∼ 1,200 mm. Topographically, the province is dominated by mountainous and hilly landscapes, with an average elevation ranging from 600 m to 1,800 m. The dominant soil types in the study area consist of Red Soil, Yellow Soil, Yellow-Brown Soil, Calcareous Soil, and Purple Soil. Among these, Yellow Soil (an acidic soil type characterized by hydrated iron oxide accumulation, typical of subtropical mountainous regions in China) occupies the largest distribution area, followed by Calcareous Soil, with *Camellia sinensis* predominantly distributed in the Yellow Soil regions. Building on prior surveys of tea garden soils in Guizhou Province, this study first collected and analyzed 146 surface soil samples from six administrative regions (sampling locations illustrated in Fig. 1). Subsequently, twelve representative tea-producing areas—two per region, namely Yinjiang Tea District (YJCQ), Shiqian Tea District (SQCQ), Leishan Tea District (LSCQ), Liping Tea District (LPCQ), Meitan Tea District (MTCQ), Fenggang Tea District (FGCQ), Kaiyang Tea District (KYCQ), Duyun Tea District (DYCQ), Guiding Tea District (GDCQ), Anshun Tea District (ASCQ), Pu’an Tea District (PACQ), and Anlong Tea District (ALCQ)—were strategically selected for the collection and analytical testing of soil profile samples. The detailed geographical coordinates of these twelve tea-producing areas are presented in Fig. 1.

 By investigating the spatial differences in the distribution of major mineral elements (aluminum (Al), Fe, calcium (Ca), magnesium (Mg), phosphorus (P), potassium (K), sulfur (S)) and trace mineral elements (Mn, Cu, Zn, molybdenum (Mo), Ge, Se, Sr) that affect the growth of tea trees, the correlation between the content levels of Zn, Se, Ge and Sr in tea garden soils and the quality of tea was explored.

### Methods for collection and pre-treatment of rock, soil, and tea samples

In July ∼August of 2024, in the six main tea-producing areas of Guizhou region, 146 soil sampling points in tea gardens were arranged to carry out surface soil (0 ∼ 20 cm) sample collection and test analysis.

In April ∼ May of 2025, based on the collection of geological, soil, and tea product data from the main tea-producing areas in Guizhou Province and the field survey of tea gardens, three representative tea gardens in each main tea-producing area were selected for soil profile excavation, considering that the thickness of the tea garden soil, planting age of 10 ∼ 20 years, tea varieties, and production management level were relatively consistent. The sampling depth was set according to the general distribution of tea root system in the soil of 0 ∼ 60 cm. In each tea garden, the soil profile was sampled in layers of 0 ∼ 20 cm, 20 ∼ 40 cm, 40 ∼ 60 cm and parent rock layer (Use A/B/C/D to represent each level respectively). In the 36 tea gardens of 12 tea-producing areas, 36 rock samples and 108 soil samples collected. Meanwhile, tender leaves samples (a mixture of new shoots with one bud and two leaves) of *Camellia sinensis* above the three soil profiles in the same sampling was collected, which the tea leaves samples were collected from multiple points of young leaves mixed together with each sampling point weighing 500 grams, and a total of 36 tea samples were collected. After the rock samples were crushed and the soil samples were air-dried, the samples were ground into powder with a mahjong grinder, sieved through a 200-mesh nylon sieve, and sealed in a polyethylene plastic bag for testing and analysis. The tender leaves of *Camellia sinensis* samples as tea samples were rinsed with water and then washed with ultra-pure water, placed in a constant temperature forced-air drying oven at 60 °C, ground into fine powder with a plant sample grinder, sieved through a 100-mesh nylon sieve, and placed in a polyethylene plastic bag, and stored for testing and analysis.

### Determination method of rock, soil and tea samples

Approximately 0.5000 g of rock or soil sample was accurately weighed and subjected to digestion using 10 mL of aqua regia (a mixture of concentrated nitric acid and hydrochloric acid at a volume ratio of 1:3). Subsequently, the concentrations of target elements in the resultant digest solutions were quantified *via* inductively coupled plasma mass spectrometry (ICP-MS, PE NexION-1100G), and the values were further converted to the element contents in the original rock and soil samples.

For tea samples, precisely 0.5000 g of tender leaves of *Camellia sinensis* was weighed and digested slowly with 10 mL of concentrated nitric acid. The element concentrations in the digests were then determined using the same ICP-MS platform (PE NexION-1100G), followed by the calculation of mineral element contents in the tea tissues.

Throughout the entire analytical procedure, method blanks, duplicate samples, and certified reference materials (CRMs) were incorporated to validate the accuracy and precision of the test results.

### Data analysis

The test data were analyzed, counted and graphed by using Microsoft Excel 201, SPSS 25.0, Origin 9.0, and the graphs were further drawn and processed by using Coreldraw 14.0 software. The variance analysis, multiple comparison analysis and regression analysis were completed on SPSS 25.0 software platform (IBM Corp., Armonk, NY, USA).

## Results

### The content range of trace elements in the soil of tea-producing areas

It can be seen from Table 1 that the contents of Mn, Cu and Zn in the surface soils of the tea gardens in Guizhou region ranged from 34.0 mg kg^−1^ to 980.0 mg kg^−1^, 3.6 mg kg^−1^ to 117.2 mg kg^−1^, and 25.0 mg kg^−1^ to 251.0 mg kg^−1^, respectively; while the contents of soil Mo and Ge ranged from 0.50 mg kg^−1^ to 5.04 mg kg^−1^, 0.06 mg kg^−1^ to 0.49 mg kg^−1^, respectively; and the contents of Se and Sr in the tea garden soils ranged from 0.11 mg kg^−1^ to 1.62 mg kg^−1^, 13.9 mg kg^−1^ to 178.3 mg kg^−1^, respectively. From the coefficient of variation of the content of trace mineral elements in the the tea garden soils, the magnitude order was Cu > Se > Mn > Mo > Sr > Zn > Ge; the variability of Cu content was the largest, followed by Se, and the variability of Ge content is the lowest.

**Table 1:** The statistical description of the content of trace mineral elements in the surface soils (0 ∼ 20 cm) of the main tea-producing areas in Guizhou region.

Area	Statistic	Mn (mg kg^−1^)	Cu (mg kg^−1^)	Zn (mg kg^−1^)	Mo (mg kg^−1^)	Ge (mg kg^−1^)	Se (mg kg^−1^)	Sr (mg kg^−1^)
Eastern region (Tongren City; *n* = 27)	Maximum	381.0	93.0	132.0	4.66	0.49	0.43	140.0
	Minimum	31.0	7.5	18.0	0.23	0.07	0.11	13.9
	Average	249.0	29.9	76.4	2.11	0.21	0.25	44.8
	CV(%)	32.69	73.28	24.85	60.19	47.09	33.60	61.63
Southeast region (Qiandongnan State; *n* = 21)	Maximum	957.0	77.7	95.0	3.61	0.34	0.45	108.5
	Minimum	142.0	10.5	22.0	0.72	0.07	0.12	19.5
	Average	540.7	26.8	73.3	1.64	0.17	0.25	46.8
	CV(%)	50.97	68.18	25.24	50.55	42.20	39.84	47.53
South region (Qiannan State; *n* = 27)	Maximum	324.0	81.3	153.0	2.97	0.47	0.85	87.4
	Minimum	34.0	3.6	25.0	0.15	0.06	0.25	26.5
	Average	158.2	28.6	69.9	1.47	0.17	0.51	58.2
	CV(%)	55.77	76.89	48.19	54.92	66.47	32.16	25.93
Central region (Guiyang-Anshun City; *n* = 22)	Maximum	980.0	107.0	213.0	5.31	0.38	1.43	153.5
	Minimum	88.0	11.4	42.0	1.12	0.07	0.28	26.0
	Average	513.1	62.2	126.0	2.64	0.20	0.71	67.5
	CV(%)	51.46	50.59	46.90	50.38	55.96	45.97	43.56
Northwest region (Zunyi City; *n* = 29)	Maximum	932.0	124.9	251.0	5.97	0.38	1.62	103.5
	Minimum	123.0	16.7	68.0	0.32	0.13	0.35	20.3
	Average	471.6	52.9	137.7	2.12	0.23	0.74	38.3
	CV(%)	58.86	71.27	37.76	67.93	39.04	53.51	53.00
Southwest region (Qianxinan State; *n* = 20)	Maximum	532.0	117.2	209.0	5.87	0.46	1.25	178.3
	Minimum	87.0	16.7	46.0	0.96	0.06	0.38	30.7
	Average	252.1	51.5	98.1	3.36	0.24	0.74	86.9
	CV(%)	51.01	69.32	52.50	50.30	45.96	39.25	54.43
Guizhou region (*n* = 146)	Maximum	980.0	148.5	251.0	5.97	0.49	1.62	178.3
	Minimum	31.0	3.6	18.0	0.15	0.06	0.11	13.9
	Average	358.6	44.2	97.4	2.18	0.20	0.53	55.4
	CV(%)	69.69	77.60	50.82	63.30	49.75	62.41	56.86

It can be seen from the average content of Mn, Cu, Zn, Mo, Ge, Se and Sr in the surface soil of tea gardens in the above six sampling areas that the soil Mn content of the northwest tea area was the highest, and the soil Mn content of the southern tea area was the lowest; the soil Cu content of the southwest tea area was the highest, and the soil Cu content of the southeast tea area was the lowest; the soil Zn content of the northwest tea area was the highest, and the soil Zn content of the southern tea area was the lowest; the soil Mo content of the southwest tea area was the highest, and the soil Mo content of the tea area was the lowest; the soil Ge content of the southwest tea area was the highest, and the soil Ge content of the southeast or southern tea areas was the lowest; the soil Se content of the northwest or southwest tea areas was the highest, and the soil Se content of the eastern or southeast tea areas was the lowest; the soil Sr content of the southwest area was the highest, and the soil Sr content of the northwest tea area was the lowest. This show that the content change of trace mineral elements in the tea garden soils had obvious regional differences in Guizhou region. It is necessary to further study the spatial distribution characteristics of trace mineral elements in tea garden soil and its correlation with tea quality.

### The accumulation characteristics of trace elements in the soil of tea-producing areas

Based on the results of the survey of the tea garden surface soils in the six areas of Guizhou region in the foregoing part, two typical tea-producing areas were further selected in each area for soil profile sample collection and test analysis. The 36 tea gardens of 12 tea-producing areas in Guizhou region were distributed on the soils formed by the weathering products different strata of sedimentary rock and metamorphic rock. The statistical analysis results of the contents of various mineral in the soil profiles (divided into layers A, B, C) and parent rock layer (D) of 36 tea gardens in the survey area are shown in Tables 2 and 3.

**Table 2:** The statistical description of the content of major mineral elements in the soils of the main tea-producing areas in Guizhou region (*n* = 36).

Layer	Statistic	Al (g kg^−1^)	Fe (g kg^−1^)	Ca (g kg^−1^)	Mg (g kg^−1^)	P (g kg^−1^)	K (g kg^−1^)	S (g kg^−1^)
Soil-A (0∼20 cm)	Maximum	108.0	104.5	1.90	7.51	9.60	41.0	1.32
	Minimum	21.3	10.6	0.21	1.30	0.96	4.8	0.10
	Average	70.0	48.7	0.87	3.89	5.05	14.5	0.57
	CV(%)	35.27	52.27	95.64	41.15	43.00	56.45	44.92
Soil-B (20∼40 cm)	Maximum	118.0	99.7	1.32	8.12	8.10	29.3	1.19
	Minimum	22.4	13.8	0.20	2.20	1.22	6.1	0.12
	Average	76.5	49.3	0.63	4.36	3.80	15.4	0.48
	CV(%)	35.25	49.13	71.95	38.75	52.34	43.82	56.96
Soil-C (40∼60 cm)	Maximum	115.5	108.0	2.30	8.69	9.15	41.6	0.93
	Minimum	22.6	11.0	0.12	1.71	1.20	5.4	0.10
	Average	80.6	53.3	0.60	4.58	3.61	17.3	0.39
	CV(%)	32.75	49.76	71.67	43.32	54.23	49.93	63.50
Rock-D	Maximum	99.5	82.5	4.60	21.6	7.72	47.1	1.50
	Minimum	9.6	9.4	0.10	0.30	0.20	1.4	0.10
	Average	54.7	31.4	1.27	6.5	2.73	17.7	0.28
	CV(%)	46.04	53.54	96.14	73.95	68.19	73.35	109.0

**Table 3:** The content range of trace mineral elements in the soils of the different tea-producing areas in Guizhou region (*n* = 3).

Sampling point	Level	Mn (mg kg^−1^)	Cu (mg kg^−1^)	Zn (mg kg^−1^)	Mo (mg kg^−1^)	Ge (mg kg^−1^)	Se (mg kg^−1^)	Sr (mg kg^−1^)
YJCY	A	298.3 ± 135.2	47.1 ± 33.1	75.7 ± 9.5	1.27 ± 0.33	0.606 ± 0.292	0.337 ± 0.104	51.9 ± 30.6
	B	394.7 ± 50.6	19.9 ± 8.0	74.0 ± 17.4	1.10 ± 0.43	0.314 ± 0.064	0.260 ± 0.050	40.8 ± 32.6
	C	267.3 ± 91.9	26.5 ± 2.5	59.0 ± 32.1	0.92 ± 0.51	0.454 ± 0.280	0.280 ± 0.050	58.7 ± 11.8
	D	334.7 ± 39.5	35.9 ± 18.3	58.2 ± 16.7	0.41 ± 0.28	0.234 ± 0.070	0.267 ± 0.038	16.0 ± 9.6
SQCY	A	323.7 ± 61.2	14.3 ± 3.5	67.5 ± 18.8	1.51 ± 0.38	0.120 ± 0.034	0.270 ± 0.458	140.7 ± 4.5
	B	288.0 ± 48.2	15.0 ± 4.4	72.7 ± 30.2	1.44 ± 0.53	0.146 ± 0.064	0.303 ± 0.114	143.7 ± 2.4
	C	303.3 ± 187.4	18.7 ± 8.5	78.7 ± 44.1	1.57 ± 0.49	0.120 ± 0.034	0.273 ± 0.047	138.1 ± 2.6
	D	198.7 ± 75.5	19.6 ± 4.2	41.2 ± 18.2	0.80 ± 0.24	0.414 ± 0.170	0.223 ± 0.035	115.5 ± 9.4
LSCY	A	451.0 ± 283.7	12.5 ± 4.4	60.0 ± 24.0	1.61 ± 0.52	0.166 ± 0.102	0.357 ± 0.025	27.1 ± 7.7
	B	447.3 ± 344.5	11.8 ± 2.3	61.0 ± 34.1	1.43 ± 0.62	0.214 ± 0.90	0.350 ± 0.053	27.6 ± 10.0
	C	317.6 ± 99.7	15.2 ± 2.7	69.7 ± 30.7	1.79 ± 0.67	0.286 ± 0.122	0.277 ± 0.040	29.5 ± 6.0
	D	285.7 ± 117.5	14.5 ± 6.3	54.9 ± 5.6	0.34 ± 0.12	0.180 ± 0.040	0.276 ± 0.035	46.8 ± 5.1
LPCY	A	244.0 ± 90.3	25.7 ± 3.6	72.3 ± 10.0	1.32 ± 0.83	0.334 ± 0.110	0.317 ± 0.090	36.1 ± 5.6
	B	354.0 ± 194.9	27.3 ± 4.2	71.3 ± 18.9	1.35 ± 0.13	0.300 ± 0.100	0.327 ± 0.108	36.0 ± 5.4
	C	327.7 ± 205.9	33.8 ± 14.0	89.7 ± 10.1	1.54 ± 0.28	0.326 ± 0.092	0.313 ± 0.111	32.0 ± 12.1
	D	191.7 ± 50.8	19.1 ± 8.7	49.8 ± 13.4	0.16 ± 0.06	0.200 ± 0.088	0.263 ± 0.015	61.2 ± 12.5
GDCY	A	36.0 ± 17.7	5.3 ± 2.2	31.3 ± 1.5	0.98 ± 0.40	0.346 ± 0.030	0.457 ± 0.091	70.7 ± 33.6
	B	42.67 ± 7.1	6.2 ± 2.4	41.7 ± 5.5	0.92 ± 0.26	0.466 ± 0.090	0.473 ± 0.040	93.6 ± 42.4
	C	35.33 ± 29.4	6.7 ± 2.9	36.0 ± 6.1	0.70 ± 0.26	0.514 ± 0.050	0.527 ± 0.083	114.4 ± 25.9
	D	58.9 ± 5.3	4.3 ± 0.9	7.3 ± 1.2	0.49 ± 0.08	0.194 ± 0.100	0.343 ± 0.031	56.2 ± 23.5
DYCY	A	763.3 ± 159.5	22.9 ± 4.9	120.0 ± 44.6	1.57 ± 1.08	0.660 ± 0.290	0.840 ± 0.089	65.9 ± 11.2
	B	735.0 ± 252.5	19.1 ± 3.0	102.0 ± 8.5	2.01 ± 1.53	0.566 ± 0.360	0.833 ± 0.051	68.1 ± 4.1
	C	572.0 ± 381.1	18.9 ± 2.9	105.7 ± 29.7	1.92 ± 1.44	0.734 ± 0.230	0.820 ± 0.176	58.6 ± 30.4
	D	209.7 ± 120.6	19.7 ± 8.3	74.4 ± 17.3	0.27 ± 0.05	0.920 ± 0.124	0.650 ± 0.066	71.8 ± 9.6
KYCY	A	699.3 ± 142.1	52.1 ± 22.0	83.7 ± 10.6	2.57 ± 0.30	0.360 ± 0.040	1.107 ± 0.117	50.1 ± 9.9
	B	773.7 ± 112.5	65.2 ± 23.9	86.5 ± 6.6	3.28 ± 0.82	0.280 ± 0.040	1.087 ± 0.153	50.9 ± 11.3
	C	677.0 ± 48.1	65.44 ± 25.4	92.8 ± 11.1	3.52 ± 0.51	0.286 ± 0.064	1.060 ± 0.151	49.6 ± 9.3
	D	466.3 ± 182.2	51.1 ± 40.9	76.5 ± 7.3	0.44 ± 0.17	0.606 ± 0.160	0.757 ± 0.126	70.2 ± 26.0
ASCY	A	182.3 ± 46.9	77.9 ± 20.0	68.0 ± 23.7	3.61 ± 1.19	0.700 ± 0.106	0.543 ± 0.064	90.6 ± 15.1
	B	116.0 ± 25.4	73.4 ± 13.4	72.7 ± 22.8	4.95 ± 2.26	0.574 ± 0.190	0.607 ± 0.075	83.5 ± 29.7
	C	137.3 ± 38.4	86.5 ± 7.3	73.7 ± 9.0	3.90 ± 1.26	0.680 ± 0.190	0.597 ± 0.097	63.4 ± 12.4
	D	51.33 ± 11.0	12.7 ± 0.4	6.3 ± 1.4	1.39 ± 0.92	0.100 ± 0.010	0.383 ± 0.031	34.6 ± 22.1
FGCY	A	602.7 ± 80.8	22.7 ± 5.8	113.2 ± 32.8	1.27 ± 0.74	0.474 ± 0.338	1.250 ± 0.217	62.8 ± 19.2
	B	684.0 ± 95.2	30.7 ± 7.2	122.3 ± 39.4	1.72 ± 1.13	0.594 ± 0.440	0.937 ± 0.123	51.0 ± 2.5
	C	782.3 ± 223.0	32.4 ± 6.1	129.0 ± 37.6	1.43 ± 0.86	0.594 ± 0.474	0.907 ± 0.191	52.0 ± 7.5
	D	363.3 ± 154.4	37.7 ± 8.6	103.2 ± 7.6	0.41 ± 0.25	0.780 ± 0.150	0.757 ± 0.117	90.2 ± 13.2
MTCY	A	205.7 ± 72.0	23.7 ± 9.3	79.4 ± 4.5	1.67 ± 0.15	0.286 ± 0.076	0.723 ± 0.266	41.0 ± 14.5
	B	185.7 ± 61.8	26.6 ± 15.6	89.5 ± 9.2	1.31 ± 0.28	0.306 ± 0.050	0.703 ± 0.230	30.3 ± 7.1
	C	241.3 ± 31.1	30.8 ± 23.0	91.5 ± 3.3	1.21 ± 0.15	0.374 ± 0.254	0.690 ± 0.156	27.4 ± 2.5
	D	57.00 ± 11.5	12.6 ± 4.6	39.6 ± 30.9	0.46 ± 0.06	0.240 ± 0.080	0.350 ± 0.075	66.5 ± 18.9
ALCY	A	344.0 ± 206.6	45.2 ± 18.9	80.5 ± 6.9	3.98 ± 0.43	0.440 ± 0.184	0.583 ± 0.097	144.4 ± 8.5
	B	532.3 ± 399.3	53.6 ± 29.3	90.7 ± 12.0	2.48 ± 0.72	0.526 ± 0.326	0.497 ± 0.068	145.8 ± 17.4
	C	329.0 ± 245.5	54.8 ± 28.6	93.4 ± 11.7	2.58 ± 0.59	0.526 ± 0.340	0.550 ± 0.070	135.5 ± 8.9
	D	558.3 ± 117.7	83.0 ± 13.6	76.0 ± 9.8	1.72 ± 0.95	0.266 ± 0.110	0.353 ± 0.042	108.4 ± 13.5
PACY	A	322.3 ± 190.5	108.6 ± 11.5	80.3 ± 28.4	3.94 ± 0.55	0.680 ± 0.334	0.937 ± 0.159	115.8 ± 15.5
	B	457.3 ± 369.7	132.8 ± 36.0	100.0 ± 7.2	4.01 ± 0.57	0.714 ± 0.128	0.920 ± 0.270	139.5 ± 10.0
	C	380.3 ± 274.9	138.8 ± 56.9	98.7 ± 18.5	4.45 ± 0.45	0.740 ± 0.144	0.847 ± 0.191	131.7 ± 30.2
	D	377.7 ± 150.5	89.0 ± 12.5	74.0 ± 5.2	1.44 ± 0.25	0.446 ± 0.062	0.687 ± 0.036	129.4 ± 9.4

**Notes.**

A\B\C\D was 0∼20 cm soil layer, 20∼40 cm soil layer, 40∼60 cm soil layer and parent rock layer, respectively. The data in the table are the mean ± standard deviation.

It can be seen from Table 2 that the contents of Al and Fe in the topsoil (0 ∼ 20 cm) of the tea gardens in Guizhou region ranged from 21.3 g kg^−1^ to 108.0 g kg^−1^, 10.6 g kg^−1^ to 104.5 g kg^−1^, respectively; while the contents of soil Ca and Mg ranged from 0.21 g kg^−1^ to 1.90 g kg^−1^, 1.30 g kg^−1^ to 7.51 g kg^−1^, respectively; and the contents of P, K and S in the tea garden soils ranged from 0.96 g kg^−1^ to 9.60 g kg^−1^, 4.8 g kg^−1^ to 41.0 g kg^−1^ and 0.10 g kg^−1^ to 1.32/kg, respectively. From the coefficient of variation of the content of major mineral elements in the the tea garden soils, the magnitude order was Ca > S > P > Fe > K > Mg > Al; the variability of Ca content was the largest, followed by S, and the variability of Al content is the lowest. Compared with parent rock strata, the contents of Al, Fe, P and S in the soils showed a significant increase, while the contents of Ca, Mg and K in the soils showed a significant decrease.

It can be seen from Table 3 that the Mn content in the surface soil (A, 0 ∼ 20 cm), middle soil (B, 20 ∼ 40 cm) and lower soil layer (C, 40 ∼ 60 cm) of the 36 tea gardens in Guizhou region was the range of 17.0 mg kg^−1^ ∼ 940.0 mg kg^−1^, 35.0 mg kg^−1^ ∼ 985.0 mg kg^−1^, 15.0 mg kg^−1^ ∼ 969.0 mg kg^−1^, respectively; among them, the soil Mn content of Kaiyang tea area was the highest, and the soil Mn content of Ansun tea area was the lowest. The content of Cu in the surface soil, middle soil and lower soil of the tea gardens was in the range of 3.8 mg kg^−1^ ∼ 119.0 mg kg^−1^, 4.6 mg kg^−1^ ∼ 173.5 mg kg^−1^, 4.0 mg kg^−1^ ∼ 202.0 mg kg^−1^, respectively; among them, the soil Cu content of Puan tea area was the highest, and the soil Cu content of Guiding area was the lowest. The content of Zn in the surface soil, middle soil and lower soil of the tea gardens was in the range of 30.0 mg kg^−1^ ∼ 171.0 mg kg^−1^, 37.0 mg kg^−1^ ∼ 148.0 mg kg^−1^, 29.0 mg kg^−1^ ∼ 156.0 mg kg^−1^, respectively, of which the soil Zn content of Kaiyang tea area was the highest, followed by Fenggang tea area; the soil Zn content of Guiding tea area was the lowest. Also, the content of Mo in the surface soil, middle layer soil and lower layer soil of the tea gardens was in the range of 0.470 mg kg^−1^ ∼ 4.69 mg kg^−1^, 0.460 mg kg^−1^ ∼ 6.78 mg kg^−1^, 0.440 mg kg^−1^ ∼ 4.95 mg kg^−1^, respectively, of which the soil Mo content of Ansun tea area was the highest, and the soil Mo content of Guiding tea area was the lowest. The content of Ge in the surface soil, middle layer soil and lower layer soil of the tea garden was in the range of 0.040 mg kg^−1^ ∼ 0.520 mg kg^−1^, 0.050 mg kg^−1^ ∼ 0.550 mg kg^−1^, 0.040 mg kg^−1^ ∼ 0.570 mg kg^−1^, respectively, of which the soil Ge content of Puan tea area was the highest, and the soil Ge content of Shiqian tea area was the lowest.While the content of Se was in the range of 0.220 mg kg^−1^ ∼ 1.50 mg kg^−1^, 0.210 mg kg^−1^ ∼ 1.23 mg kg^−1^, 0.210 mg kg^−1^ ∼ 1.230 mg kg^−1^, respectively, of which the soil Se content of Kaiyang tea area or Fenggang tea area was the highest, followed by Puan tea area, and the soil Se content of Shiqian tea area was the lowest.

**Figure 2:**
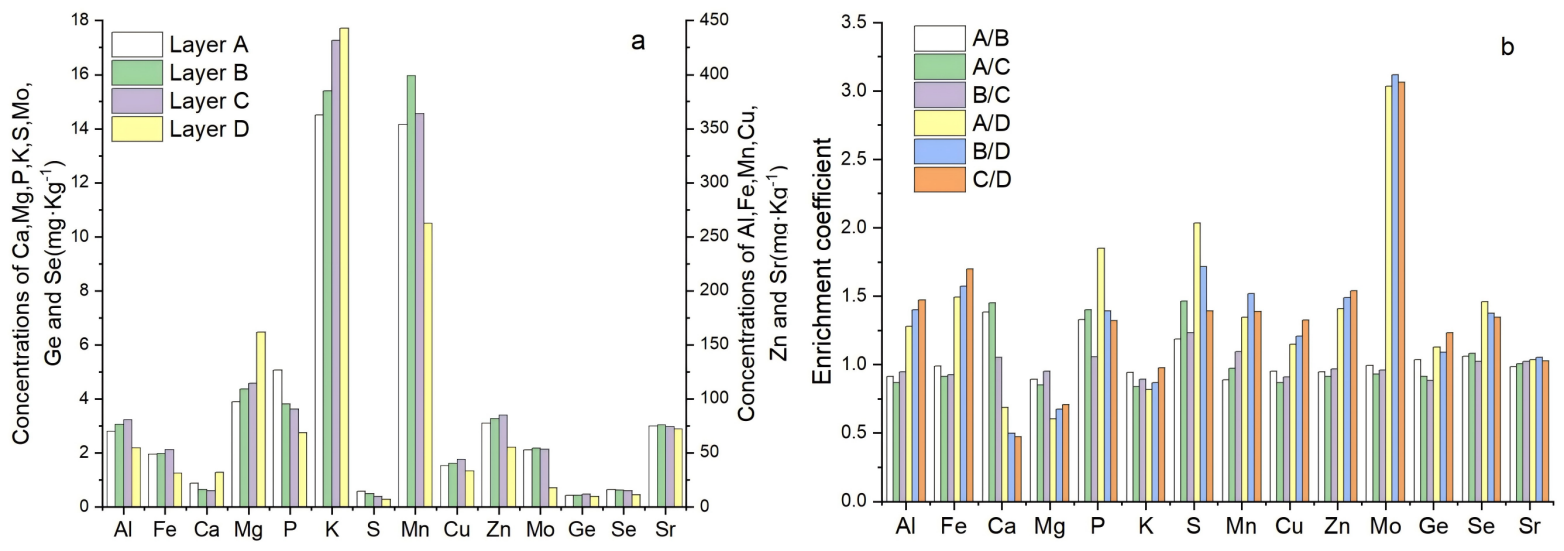
Average content (A) and enrichment coefficient (B) of mineral elements in the soil and rock of Guizhou tea garden (*n* = 12). A/B/C/D was 0 ∼ 20 cm soil layer, 20 ∼ 40 cm soil layer, 40 ∼ 60 cm soil layer and parent rock layer, respectively.

In addition, the content of Sr in the surface soil, middle layer soil and lower layer soil of the tea gardens ranged from 17.5 mg kg^−1^ ∼ 152.5 mg kg^−1^, 16.1 mg kg^−1^ ∼ 158.5 mg kg^−1^, 19.5 mg kg^−1^ ∼ 166.0 mg kg^−1^, respectively, of which the Sr content in the soil of Anlong tea area was the highest, and the Sr content in the soil of Leishan tea area was the lowest (Fig. 2). It can be seen that the distribution of trace mineral elements in tea garden soils had obvious regional, especially higher Cu, Ge, Se in the soil of Puan tea area, higher Mn, Zn, Se in the soil of Kaiyang tea area and higher Zn, Se in the soil of Fenggang tea area. Also, higher Mo content in the soil of Anshun tea area, and lower Mn content. But lower Ge, Se content in the soil of Shiqian tea area, lower Cu, Zn, Mo content in the of Guiding tea area. According to the current national standards or industry standards of China, such as “Soil selenium grade” (GB/T 4971-2024), and “Specification of land quality geochemical assessment” Ministry of Land and Resources of the People’s Republic of China (2016) _(DZ/T 0295-2016) (Zn-rich soil≥80 mg kg^−1^, Se-rich soil≥0.4 mg kg^−1^, Ge-rich soil≥1.5 mg kg^−1^, Sr-rich soil≥200 mg kg^−1^), from the soil Zn or Se content of the 36 tea gardens in Guizhou region, 69.4% of tea garden surface soils (0 ∼ 20 cm) reached the level of Se-enriched soil, but 33.3% of tea garden surface soils reached the level of Zn-Se-enriched soil. These Zn-Se-enriched soils of tea garden were mainly distributed in the three tea-producing areas, such as Duyun Tea Garden, Kaiyang Tea Garden, Fenggang Tea Garden. However, there was no tea garden soils rich in Ge or Se of the soils at survey areas.

From the perspective of the accumulation coefficient of soil mineral elements (the content of A or B or C layer/the content of parent rock), the order of the accumulation coefficient of the main mineral elements in the tea garden soils of Guizhou region was Fe > Al > S > P > K > Ca > Mg, and its average accumulation coefficient range was 1.49 ∼ 1.70, 1.28 ∼ 1.47, 1.39 ∼ 2.04, 1.32 ∼ 1.85, 0.82 ∼ 0.98 0.47 ∼ 0.69, 0.60 ∼ 0.71, respectively; while the order of the accumulation coefficient of trace elements in the tea garden soil was Mo > Zn > Mn > Se > Cu > Ge > Sr, its average accumulation coefficient was 3.03 ∼ 3.12, 1.41 ∼ 1.54, 1.35 ∼ 1.52 1.35 ∼ 1.46, 1.15 ∼ 1.32, 1.09 ∼ 1.23, 1.03 ∼ 1.05, respectively. It can be seen that the weathering process of rocks and the process of soil formation had produced leaching loss of Ca, Mg and K in the rocks, while other mineral elements had shown different degrees of precipitation and accumulation.

### Differences in the content of trace mineral elements in different tea garden soils and their dominant factors

It could be seen from the results of the multiple comparison of the content of trace mineral elements in the soils of 12 tea areas in Table 4, among the seven trace elements, the difference of Cu and Se content is the largest, followed by Zn and Sr, then by Mn, Mo, and the difference of Ge content was the smallest. The change of geological environmental conditions in different areas would have a significant impact on the content of trace mineral elements (Mn, Cu, Zn, Mo, Se, Ge, Sr) in the tea garden soils, and parent material was the main source of soil mineral elements, directly affecting the content level and distribution law of soil trace mineral elements.

It could be seen from Table 5 that the contents of Mn, Cu, Zn, Mo, Se, Sr in the soil profile layers (A, B, C) of tea gardens were positively correlated with the content of the corresponding elements in the parent rock, especially Se and Cu, followed by Zn, Mo, Sr, and then Mn. In addition, from the content change of trace mineral elements in the soil profile layer A, B, C (Table 3), the contents of Mn, Cu, Zn, Mo, Ge in the tea garden soils increased with the increase of soil depth, but the content of Se showed a downward trend with the increase of depth, which indicated that the migration and accumulation characteristics of different trace mineral elements in the process of soil development also affected content level of various trace mineral elements in the soil profile of the tea gardens. It could be seen that the variability of parent material and soil-forming process is the main factor affecting the content level of trace mineral elements in regional tea garden soils, which had a close inheritance with the parent material; and the regional geological environment conditions (atigraphic age and rock combination) played a decisive role in the influence of trace mineral elements in the soils, especially Zn and Se. The parent material and soil-making process determine whether the contents of Zn, Se and Ge in the tea garden soil could reach the content level of rich in Zn, Se, Ge.

**Table 4:** Differences in the content of trace elements in the soils of different tea-producing areas in Guizhou region (*n* = 3).

Sampling point	Mn (mg kg^−1^)	Cu (mg kg^−1^)	Zn (mg kg^−1^)	Mo (mg kg^−1^)	Ge (mg kg^−1^)	Se (mg kg^−1^)	Sr (mg kg^−1^)
YJCY	320.1 ± 66.4cd	31.2 ± 14.2d	69.6 ± 9.2f	1.10 ± 0.17cd	0.458 ± 0.146c	0.292 ± 0.040e	50.5 ± 9.0de
SQCY	305.0 ± 17.9cd	16.0 ± 2.4efg	73.0 ± 5.6ef	1.51 ± 0.06cd	0.128 ± 0.016e	0.282 ± 0.018e	140.8 ± 2.8a
LSCY	511.2 ± 107.5b	13.2 ± 1.8fg	63.6 ± 5.3f	1.61 ± 0.18cd	0.222 ± 0.060de	0.328 ± 0.044e	28.1 ± 1.3f
LPCY	308.7 ± 57.5cd	28.9 ± 4.3de	77.8 ± 10.3def	1.40 ± 0.12cd	0.320 ± 0.018d	0.319 ± 0.072e	34.7 ± 2.3ef
GDCY	38.0 ± 4.09f	6.1 ± 0.7 g	36.3 ± 5.2 g	0.87 ± 0.15d	0.442 ± 0.086c	0.486 ± 0.037d	92.9 ± 21.9b
DYCY	690.1 ± 103.2a	20.3 ± 2.2def	109.2 ± 9.5b	1.83 ± 0.23c	0.654 ± 0.084ab	0.830 ± 0.010b	64.2 ± 5.0cd
KYCY	716.7 ± 50.6a	60.9 ± 7.6c	175.3 ± 9.4a	3.12 ± 0.49b	0.308 ± 0.044d	1.087 ± 0.025a	50.2 ± 0.7de
ASCY	145.2 ± 33.8ef	79.3 ± 6.7b	71.5 ± 3.0f	4.69 ± 0.97a	0.652 ± 0.068ab	0.582 ± 0.034d	79.2 ± 14.1bc
FGCY	689.7 ± 89.3a	28.6 ± 5.2de	121.6 ± 8.0b	1.47 ± 0.23cd	0.554 ± 0.070bc	1.031 ± 0.190a	55.3 ± 6.5d
MTCY	210.9 ± 28.2de	27.0 ± 3.6de	86.8 ± 6.5cde	1.40 ± 0.24cd	0.322 ± 0.046d	0.705 ± 0.017c	32.9 ± 7.2f
ALCY	401.8 ± 113.3bc	51.2 ± 5.2c	88.2 ± 6.8cd	3.01 ± 0.84b	0.498 ± 0.050c	0.543 ± 0.043d	141.9 ± 5.6a
PACY	386.6 ± 67.7bc	126.7 ± 16.0a	93.0 ± 11.0c	4.13 ± 0.28a	0.712 ± 0.030a	0.901 ± 0.048b	129.0 ± 12.1a

**Notes.**

The data in the table are the mean ± standard deviation. The letters are the results of multiple comparison (LSD) and different letters in the same column mean significance at *p* < 0.05 level based on multiple comparisons by SSR.

**Table 5:** Correlation coefficients between the contents of trace mineral elements in the tea garden soil and the content of corresponding elements in the rock (*n* = 36).

Factor	Mn	Cu	Zn	Mo	Ge	Se	Sr
Soil-A	0.385^*^	0.620^**^	0.570^**^	0.681^**^	0.130	0.887^**^	0.539^**^
Soil-B	0.530^**^	0.689^**^	0.588^**^	0.557^**^	0.161	0.863^**^	0.566^**^
Soil-C	0.441^**^	0.633^**^	0.559^**^	0.543^**^	0.174	0.871^**^	0.544^**^

**Notes.**

A\B\C was 0∼20 cm soil layer, 20∼40 cm soil layer, and 40∼60 cm soil layer, respectively.

** indicates the level of significance (*P* < 0.01).

* indicates the level of significance (*P* < 0.05).

It could be seen from the results of correlation analysis between the average content of Zn, Se, Ge, Sr in the surface soils of 36 tea gardens and the contents of other mineral elements (Table 6), of which the content of Zn in the soil was extremely significantly positively correlated with the contents of Al, Mg, Mn in the soils; while the content of Se in the soil was extremely significantly positively correlated with the contents of Al, Fe, Mg, Mn in the soil. Also, the content of Ge in the soil was significantly positively correlated with the contents of Fe, S, Cu in the soils; while the content of Sr in the soil is extremely significantly positively correlated with the contents of Cu, Mo in the soils. At the same time, the correlation between Zn content and Se content in tea garden soils also reached the level of extremely significant correlation (*n* = 36, the correlation coefficients of A, B, C layers were 0.506, 0.510, 0.499, respectively; *P* < 0.01), indicating that there was a synergistic effect between Zn and Se in tea garden soils. However, the correlations between the contents of Zn, Se and the contents of Ge, Sr in the tea garden soils didn’t reach a significant level. It could be seen that content level of Zn, Se, Ge and Sr in the tea garden soils was not only closely related to the contents of Zn, Se, Ge and Sr in the parent material, but was also significantly affected by the content level of other mineral elements in the soil.

**Table 6:** Correlation coefficients of the contents of Zn, Ge, Se and Sr in tea garden soils with other elements (*n* = 36).

Factor	Al	Fe	Ca	Mg	K	P	S	Mn	Cu	Mo
Zn-A	0.479^**^	0.274	0.246	0.487^**^	0.033	0.046	0.185	0.628^**^	0.131	0.109
Se-A	0.345^*^	0.399^*^	0.328	0.500^**^	−0.163	0.281	0.181	0.517^**^	0.238	0.206
Ge-A	0.150	0.410^*^	0.095	0.236	0.187	0.020	0.418^*^	0.075	0.475^*^	0.295
Sr-A	−0.276	0.276	−0.054	−0.111	−0.341^*^	0.020	0.182	−0.141	0.333^*^	0.502^**^

**Notes.**

The average content of mineral elements in the A (0∼20 cm), B (20∼40cm) and C (40∼60 cm) layers of the tea garden soil was used to perform correlation analysis.

** indicates the level of significance (*P* < 0.01).

* indicates the level of significance (*P* < 0.05).

### Variation of trace mineral elements in tea from different tea-producing areas and the response of tea quality

It could be seen from Table 7 that the contents of Mn, Cu, Zn in the tea of 12 tea-producing areas in Guizhou region ranged from 390.8 mg kg^−1^ to 1,742.0 mg kg^−1^, 9.25 mg kg^−1^ to 23.90 mg kg^−1^, and 24.4 mg kg^−1^ to 97.2 mg kg^−1^, respectively. Among them, the tea from Fenggang tea-producing area had the highest Mn content, and the tea from Guiding tea-producing area had the lowest Mn content; the tea of Puan tea-producing area had the highest Cu content, and the tea of Guiding tea-producing area had the lowest Cu content; the tea from Fenggang tea-producing area the highest Zn content, and the tea from Guiding tea-producing area had the lowest Zn content. Also, the contents of Mo, Ge, Se, Sr in the soils of the 12 tea-producing areas in Guizhou region ranged from 0.021 mg kg^−1^ to 0.071 mg kg^−1^, 0.010 mg kg^−1^ to 0.083 mg kg^−1^, 0.047 mg kg^−1^ to 0.537 mg kg^−1^, 2.85 mg kg^−1^ to 16.20 mg kg^−1^, respectively, of which the tea of Leishan tea-producing area had the highest Mo content, and the tea of Puan tea-producing area and Guiding tea-producing area had the lowest Mo content; the tea from Duyun tea-producing had the highest Ge content, and the tea from Guiding tea-producing area had the lowest Ge content; the tea of Fenggang tea-producing area had the Se content, and the tea of Guiding tea-producing area had the lowest Se content; the tea from Puan tea-producing area had the highest Sr content and the tea from Guiding tea-producing area had the lowest Sr content.

**Table 7:** Variation of trace mineral elements in tea in different tea-producing areas of Guizhou region (*n* = 3).

Sampling point	Mn (mg kg^−1^)	Cu (mg kg^−1^)	Zn (mg kg^−1^)	Mo (mg kg^−1^)	Ge (mg kg^−1^)	Se (mg kg^−1^)	Sr (mg kg^−1^)
YJCY	774.2 ± 127.7de	15.2 ± 1.5abcd	38.4 ± 2.8bcd	0.047 ± 0.009b	0.067 ± 0.020a	0.103 ± 0.018efg	4.13 ± 0.27d
SQCY	846.0 ± 95.7bcde	19.4 ± 4.2ab	38.6 ± 3.4bcd	0.039 ± 0.016b	0.017 ± 0.005d	0.118 ± 0.015def	7.28 ± 1.39b
LSCY	1,076.1 ± 154.1b	14.8 ± 4.4abcd	41.2 ± 6.2b	0.363 ± 0.009bc	0.015 ± 0.004d	0.085 ± 0.007efg	4.60 ± 1.12cd
LPCY	986.7 ± 51.1bcd	13.6 ± 2.1cd	33.7 ± 6.3cd	0.047 ± 0.006ab	0.020 ± 0.005cd	0.076 ± 0.008fg	4.15 ± 0.78d
GDCY	494.7 ± 142.4f	10.5 ± 1.4d	27.5 ± 3.8d	0.031 ± 0.009b	0.014 ± 0.004d	0.058 ± 0.010 g	3.25 ± 0.35d
DYCY	935.8 ± 98.1bcd	17.9 ± 4.2abc	77.7 ± 4.3a	0.037 ± 0.005b	0.033 ± 0.007bc	0.223 ± 0.040c	4.14 ± 0.40d
KYCY	1,051.4 ± 150.0bc	17.6 ± 2.6abc	45.8 ± 7.2b	0.036 ± 0.012bc	0.018 ± 0.004d	0.373 ± 0.034b	6.55 ± 1.36bc
ASCY	812.5 ± 125.6cde	18.4 ± 3.1abc	45.3 ± 7.6b	0.059 ± 0.015ac	0.036 ± 0.007b	0.132 ± 0.021de	7.55 ± 1.43b
FGCY	1,492.6 ± 223.8a	14.2 ± 2.7bcd	86.6 ± 10.5a	0.034 ± 0.008b	0.027 ± 0.004bcd	0.492 ± 0.051a	7.87 ± 1.21b
MTCY	931.4 ± 87.7bcd	14.3 ± 2.1bcd	47.5 ± 8.8b	0.046 ± 0.010ab	0.015 ± 0.003d	0.236 ± 0.040c	8.09 ± 1.49b
ALCY	769.4 ± 129.6de	17.9 ± 1.2abc	38.7 ± 7.0bcd	0.036 ± 0.007ab	0.020 ± 0.005cd	0.160 ± 0.032d	13.80 ± 2.23a
PACY	662.8 ± 154.8ef	20.1 ± 2.2a	40.7 ± 6.2cbc	0.031 ± 0.002b	0.035 ± 0.011b	0.247 ± 0.026c	15.07 ± 1.36a

It could be seen that the contents of Mn, Zn, Se in the tea of Fenghuang tea-producing area were high, the contents Cu, Sr in the tea of Puan tea-producing area were high; while the contents of Mn, Cu, Zn, Mo, Ge, Se, Sr in the tea of Guiding tea-producing area were low. There was a significant correlation between the content level of trace mineral elements in tea and the content level of trace mineral elements in the soil. The correlation between the contents of Mn, Cu, Zn, Se, Sr in the tea and the contents of corresponding mineral elements in the soil was extremely significant (*n* = 36, R is 0.584, 0.475, 0.742, 0.851, 0.602; *P* < 0.01), especially Se, followed by Zn; and the correlation between the content of Ge in the tea and the content of Ge in the soil also reached a significant level (*n* = 36, *R* = 0.386; <0.05). Therefore, the distribution of Zn-Se-enriched tea gardens had a distinct regional nature, depending on the type of soil parent material and the content level of soil mineral elements. It could be seen from the results of the multiple comparison of the content of trace mineral elements in the tea from tea-producing areas in Table 6, there were significant differences in the contents of Mn, Cu, Zn, Mo, Ge, Se, Sr,of which the difference in Se was the largest, followed by Zn, Sr, then Mn, Ge,and the difference in the contents of Cu, Mo was lower.

From the tea Se content of the 36 tea gardens in Guizhou region, 25.0% of tea gardens reached the level of Se-enriched tea (Se, 0.25 ∼ 4.00 mg kg^−1^) in “Rich-selenium tea” by China’s Ministry of Agriculture and Rural Affairs Ministry of Agriculture of the People’s Republic of China (2002) _NY/T 600-2002; and 22.2% of tea gardens reached the level of Zn-Se-enriched tea, referring to the range of Zn or Se content in tea of “Geographical Indication Products-Fenggang Zinc Selenium Tea” (Se, 0.25 ∼ 3.50 mg kg^−1^; Zn, 40 ∼ 100 mg kg^−1^). These tea gardens were distributed in the five tea-producing areas (DuyunTea Area, Kaiyang Tea Area, Fenggang Tea Area, MeitanTea Area, Puan Tea Area), especially, which the range of tea Se content in Fenggang Tea Area was 0.436 ∼ 0.537 mg kg^−1^, and the range of tea Zn content in Fenggang Tea Area was 76.3 mg kg^−1^ ∼ 97.2 mg kg^−1^. Moreover, the content range of Ge in tea of the 12 tea-producing was 0.010 mg kg^−1^ ∼ 0.047 mg kg^−1^, and the content range of Sr was 2.36 mg kg^−1^ ∼ 21.40 mg kg^−1^; at present, China has not yet formulated relevant industry standards.

## Discussion

### Regional distribution of the tea garden soils rich in zinc or selenium and its dominant factors

The content of Zn or Se in tea garden soils showed obvious spatial heterogeneity in Guizhou region, and only some tea garden soils reached the level of Zn-enriched soil or Se-enriched soil. In this study, the content of Zn or Se in the tea garden soils was significantly positively correlated with the content of Zn or Se in parent rock, and the type of parent rock had a significant effect on the content of Zn and Se in tea garden soils. The parent material of the tea garden soils with high zinc or selenium content in the investigation area was mostly sedimentary rock weathering products such as gray-greenale shale and sandstone intercalation, sandstone and shale intercalation (Fenggang tea area, Meitan tea area), and mudstone, shale and sandstone intercalation of the Permian and Triassic strata (Puan tea area, Anlong tea area); while the parent material of the tea garden soils with low selenium content was mostly metamorphic rock weathering products such as Cambrian slate and metamorphic sandstone (Yingjiang tea area, Shiqian tea area, Leishan tea area, Liping tea area), and Silurian fine-grained sandstone and quartz sandstone (Duyun tea area, Guiding tea area). The rock combination of different areas obviously affected the geochemical process of soil mineral elements and the content level of Zn and Se in the soils. Judging from the research results of tea-producing areas with a relatively high Se content in the tea that had been reported in China so far, the Se content in the soils varies greatly in different regions, and the Se content of the soils was mainly affected by parent material and climatic conditions (Dong et al., 2022); most tea garden soils with high Se content (≥0.4 mg kg^−1^) are distributed in the southern tea areas in China, for example, the Se content of the tea garden soil on Tianba Village, Fenggang County of Guizhou Province is in the range 0.45 mg kg^−1^∼1.97 mg kg^−1^, its parent material is the weathering product of gray-greenale shale and sandstone intercalation (Liu, Xu & Shao, 2012); the Se content in the tea from Puan Tea Farm of Puan County was 0.28 mg kg^−1^ ∼ 0.33 mg kg^−1^, the tea garden soils mostly developed from mudstone weathering products, and the Se content of tea garden soils reached 0.59 mg kg^−1^ ∼ 3.07 mg kg^−1^ (Ren, Zhao & Chen, 2012); also, the ability of soil rich in Se was: shale > Triassic sandstone > Jurassic sandstone in the Wanyuan area of Sichuan Province (Zhang, 2014). It could be seen that the stratum age and the rock combination were the dominant factors affecting the content level of Zn and Se in the tea garden soils of Guizhou region. The content of Zn or Se in the tea garden soils was not only affected by the type of parent rock and the content level of other mineral elements, but also there was a significant synergistic effect between Zn and Se (Zhang et al., 2007; Duan & Chen, 2018), thus jointly promoting the obvious enrichment of zinc and selenium in the tea garden soils.

However, the content level of Zn and Se in the soil developed from the same parent rock was also affected by factors such as soil acidity andinity, organic matter, texture, moisture, and fertilization management (Han et al., 2018; Ma & Lin, 2019); Se content of the soil was significantly negatively correlated with pH value, and significantly positively correlated with organic matter content (Jiang, 2011), and the total Se content of the soil was positively correlated with the content of alkaline N, available K, and available P (Zhao, 1991). Also, there was a significant correlation between the contents of Zn and Se in the tea garden soil or tea (Song, Pan & He, 2018; Zhang et al., 2020a; Zhang et al., 2020b), and sufficient soil nutrition was beneficial to the improvement of Se content in tea (Ma & Lin, 2019); there was a significant correlation between the Se content in tea and the content of soil organic matter, alkaline N, Zn, and S or Zn content in tea (Zhang et al., 2020a; Zhang et al., 2020b); drought stress will cause the rate of absorption Se from soil by *Camelliasinensis* to decrease (Zhou et al., 2015). Therefore, the tea garden soils with high Zn and Se content do not necessarily produce Zn-Se-enriched tea, the production of Zn-Se-enriched tea was not only closely related to the level of Zn and Se content in the tea garden soils, but also affected by the physicochemical properties of the soil. When the content of available zinc or selenium in the soil reached a relatively high level, fully meeting the absorption and accumulation requirements of tea plants, it could meet the production standards of zinc and selenium-rich tea.

### The biological enrichment characteristics of zinc and selenium in tea and ecological management of tea garden

**Figure 3:**
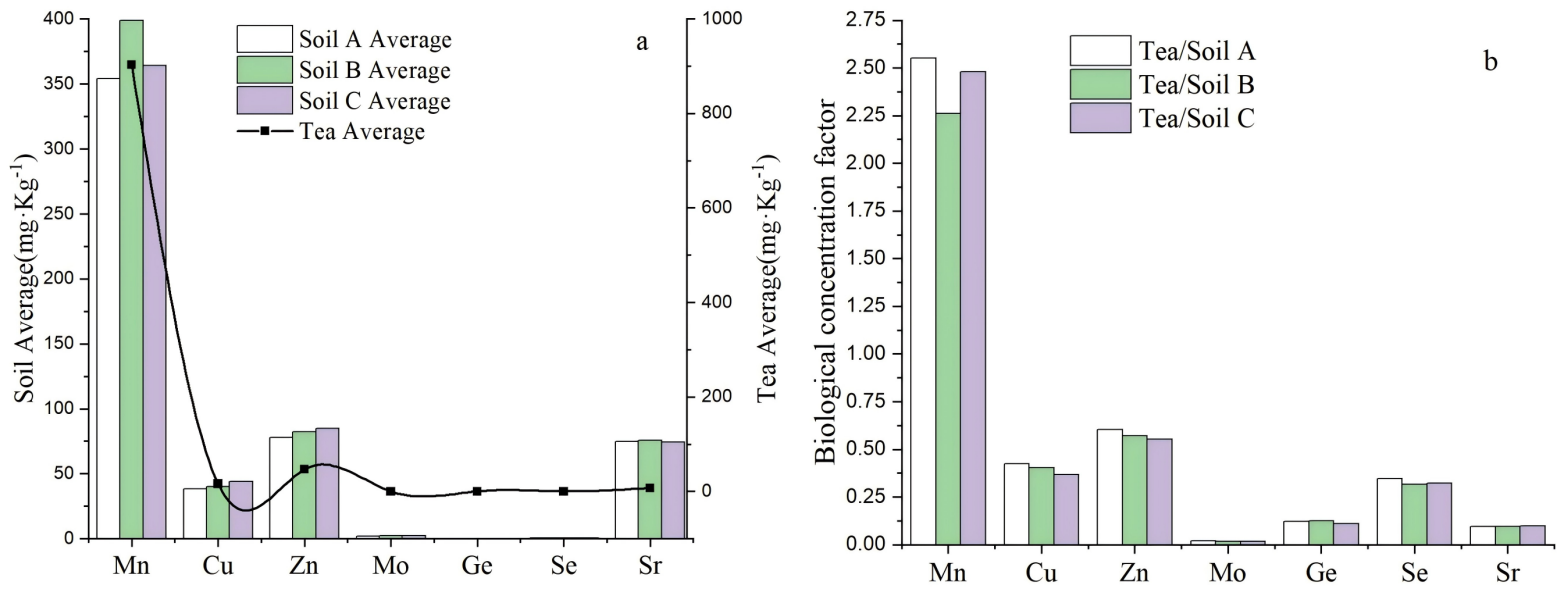
Contents (A) and bioaccumulation coefficients (B) of trace mineral elements in tea leaves and soil profile layers from the Guizhou region. A/B/C was 0 ∼ 20 cm soil layer, 20 ∼ 40 cm soil layer and 40 ∼ 60 cm soil layer, respectively.

The enrichment of different trace mineral elements in tea leaves showed obvious variability, which affects the quality and value of tea. From the results of biological concentration factor (BCF) of trace mineral elements (Mn, Cu, Zn, Mo, Ge, Se, and Sr) in tea leaves in 12 tea-producing areas of Guizhou region (Fig. 3), the order of BCF of trace mineral elements in tea leaves was Mn > Zn > Cu > Se > Sr > Ge > Mo; the average BCF value of Mn in tea leaves in the survey area ranged from 2.26 to 2.55 (BCF > 1), indicating that the ability of tea leaves to accumulate Mn was extremely strong, presenting a strong accumulation feature; and the average BCF value of Cu, Zn, Se, and Ge in tea leaves ranged from 0.111 to 0.603 (0.1 < BCF < 1), presenting a moderate accumulation feature; the average BCF value of Mo and Sr in tea leaves ranged from 0.018 to 0.097 (0.01 < BCF < 0.1), presenting a weak accumulation feature. It could be seen that the absorption of Zn or Se in the soils by *Camelliasinensis* has a more obvious enrichment property.

The Zn or Se content level in tea leaves was affected by a variety of environmental factors, and there is an obvious difference in the amount of Zn or Se absorbed and accumulated by *Camelliasinensis* in the same area (Zhang et al., 2007; Duan & Chen, 2018). Since the zinc and selenium in the soil, that could be absorbed and utilized by *Camelliasinensis,* were effective zinc and selenium, so the content of bioavailable Zn or Se in tea garden soils was the important factor to determine the production of Zn-Se-enriched tea. There was a significant correlation between the effective Zn or Se content of the soils and soil pH, soil type, organic matter, clay, soil redox status, and soil nutrients (Liao et al., 2007; Wang et al., 2018; Zhu et al., 2020). Soil acidification was a common phenomenon in tea gardens of the south China, with low pH value and strong soil acidity, and selenium in the soil mainly in the form of selenite (SeO_3_^−^), which was easily adsorbed or chelated by semi-oxides, clay minerals, and organic matter, thus reduced the availability of selenium in the soil. By analyzing the contents of total Se and effective Se in the soils of 60 typical tea gardens in Fujian Province and their influencing factors, it was found that the total Se and effective Se in the tea garden soils were significantly positively correlated with the content of soil organic matter, and the content of availability Se in tea garden soils was mainly affected by soil organic matter and total nitrogen (Yu et al., 2020), while the available K content of the soils had significantly positively correlated with the Se content of tea. Moreover, several studies have found that soil organic matter had a dual effect on the availability of selenium in soil.On the one hand, organic matter had a strong fixation capacity for soil selenium, which will reduce the availability of soil selenium, on the other hand, the increase of organic matter promoted the accumulation of organic Se, while organic Se will form soluble small organic Se through mineralization, it was easy to be absorbed by *Camelliasinensis* (Wang et al., 2016; Chang et al., 2019). Meanwhile, soil organic matter was positively correlated with the effective Zn content in soil, while soil organic matter could activate insoluble zinc in the soil, resulting in an increase in the content of exchangeable zinc (Liu et al., 2015). Moreover, there was an obvious synergistic effect between zinc and selenium in the soil, and there was a significant positive correlation between the content of soil available Zn and the content of soil total Se, the content of Se or Zn in tea, and there was also a significant positive correlation between the content of soil total Se and the content of Se or Zn in the tea (Zhang et al., 2024). It could be seen that the soil pH of tea garden directly affects the forms of Zn and Se in soils, transformation and biological availability of Zn and Se in soil; while the content of organic matter had a significant effect on the content of soil effective Zn or Se and the contents of Zn or Se in tea; So these conclusions have important guiding significance for the ecological management of tea gardens.

The management measures such as soil acidity prevention and control, soil acidity adjustment, organic fertilizer application and balanced fertilization could be taken to improve the contents of availability Zn and Se in the soils, and promote the absorption of Zn and Se by *Camelliasinensis* and enhanced the content of Zn and Se in the tea. However, the physicochemical properties of tea gardens soil with different parent materials, soil types, climatic conditions and *Camelliasinensis* planting years vary greatly, and there were large differences in the availability of Zn and Se in tea garden soils and the biological enrichment property. Moreover, there were differences in the management measures and fertilization levels among different tea-producing areas, it also leaded to regional differences in the contents of Zn or Se in tea garden soils and tea leaves. It was worthwhile to further study the correlation between the content of Zn or Se in tea and the soil theochemical properties in different tea-producing areas, to further explore the mechanism of *Camelliasinensis* absorption and enrichment of Zn and Se, to rationally select Zn-Se-enriched soils for planting *Camelliasinensis* so to develop characteristic tea-industry, and to fully use the soil testing and formula fertilization technology to regulate the ability of tea garden soil for supply bioavailable Zn and Se, which was conducive to improving the tea quality and industrial value of Zn-Se-enriched tea.

## Conclusions

The content of trace mineral elements in the soils of different tea gardens in Guizhou region showed obvious spatial variability, of which Cu and Se were the most variable, followed by Zn and Sr, and then Mn and Mo, and the lowest was Ge. The Se content in the surface soil (0∼20 cm) of tea garden in the investigation area ranged from 0.23 mg kg^−1^ to 1.50 mg⋅ kg^−1^, while the Zn content ranged from 33.0 to 105.6 mg kg^−1^, with 69.4% of the tea garden soils reaching the level of Se-enriched soil and 33.3% of the tea garden soils reaching the level of Zn-Se-enriched soil. But the tea garden soils of rich in Ge or Sr have not been found.

The change of geological environmental conditions in different regions would have a significant impact on the contents of trace mineral elements (Mn, Cu, Zn, Mo, Se, Ge, Sr) in tea garden soils. The contents of Mn, Cu, Zn, Mo, Se, Sr in tea garden soils (0 ∼20 cm, 20 ∼40 cm, 40 ∼60 cm) was significantly positively correlated with the contents of corresponding elements in parent rock, especially Se and Cu. The rock combination was the dominant factors affecting the content level of Zn and Se in tea garden soils in Guizhou region. The contents of Zn and Se in tea garden soils were not only by the type of parent rock and the contents of other mineral elements, but also had a significant synergistic effect between Zn and Se, thus promoting the obvious enrichment of Zn, Se in tea garden soils.

The contents of trace mineral elements (Mn, Cu, Zn, Mo, Ge, Se, Sr) in tea leaves from different tea gardens were significant differences, among which the difference in Se content was the largest, followed by Zn and Sr. The order of biological accumulation coefficient of trace mineral elements in tea leaves was: Mn > Zn > Cu > Se > Sr > Ge > Mo. *Camelliasinensis* had a significant enrichment characteristic for the absorption of Zn, Se in the soils, and the correlation between the content of Zn or Se in tea leaves and the content of Zn or Se in the soils was extremely significant, especially for Se. The content of Se and Zn in tea leaves in the survey areas ranged from 0.05 mg kg^−1^ to 0.54 mg kg^−1^ and from 24.4 mg kg^−1^ to 97.2 mg kg^−1^, respectively, with 25.0% of tea gardens meeting the requirements for the production of Se-enriched tea and 22.2% of tea gardens meeting the requirements for the production of Zn-Se-enriched tea. The distribution of tea garden soils rich in Zn and Se was characterized by obvious regionality, the content of Zn or Se in tea was mainly affected by the content level of Zn or Se in the soils. However, the tea garden soil with high zinc and selenium content does not necessarily produce the Zn-Se-enriched tea, and is also significantly with other soil factors.

##  Supplemental Information

10.7717/peerj.21210/supp-1 Supplemental Information 1 Analysis data of soil profiles and tea-leaves samples from 12 representative tea gardens in Guizhou Province

## Additional Information and Declarations

### Data Availability

The following information was supplied regarding data availability: The raw data is available in the Supplemental File.
