# Supplementary material for: Distribution characteristics of trace mineral elements in tea garden soils of Guizhou region and their biological accumulation and response to tea quality
Source: PeerJ. 2026 May 20;14:e21210. doi: 10.7717/peerj.21210 (PMC13198199; doi:10.7717/peerj.21210)
Supplement: Supplemental Information 1 [file peerj-14-21210-s001.docx]

**Supplementary data**

**Table 1-1 Analysis data of topsoil samples from 146 tea gardens in Guizhou Province(mg·kg^-1^)**

| Region | Mn | Cu | Zn | Mo | Ge | Se | Sr |
| --- | --- | --- | --- | --- | --- | --- | --- |
| Eastern region  （Tongren City;  n=27） | 230 | 46.8 | 78 | 1.26 | 0.28 | 0.39 | 42.2 |
|  | 210 | 33.2 | 61 | 1.15 | 0.28 | 0.15 | 49.8 |
|  | 340 | 89.5 | 95 | 1.25 | 0.24 | 0.19 | 33.9 |
|  | 225 | 9.2 | 132 | 0.94 | 0.49 | 0.13 | 93.0 |
|  | 327 | 19.3 | 92 | 0.70 | 0.21 | 0.15 | 13.9 |
|  | 229 | 48.6 | 80 | 2.69 | 0.20 | 0.23 | 17.5 |
|  | 131 | 12.2 | 28 | 4.36 | 0.08 | 0.11 | 20.1 |
|  | 350 | 93.0 | 84 | 0.87 | 0.30 | 0.18 | 90.5 |
|  | 224 | 47.4 | 91 | 4.66 | 0.23 | 0.43 | 43.4 |
|  | 329 | 18.6 | 89 | 1.08 | 0.21 | 0.19 | 60.1 |
|  | 216 | 18.3 | 72 | 1.06 | 0.21 | 0.29 | 58.1 |
|  | 149 | 18.1 | 61 | 1.02 | 0.22 | 0.36 | 63.0 |
|  | 155 | 12.8 | 73 | 2.60 | 0.19 | 0.32 | 25.6 |
|  | 207 | 13.6 | 81 | 2.95 | 0.21 | 0.27 | 24.1 |
|  | 232 | 14.0 | 84 | 2.92 | 0.21 | 0.26 | 25.1 |
|  | 224 | 15.8 | 121 | 1.63 | 0.17 | 0.22 | 41.0 |
|  | 328 | 16.9 | 151 | 1.79 | 0.14 | 0.28 | 44.1 |
|  | 349 | 18.4 | 92 | 1.87 | 0.12 | 0.24 | 48.3 |
|  | 381 | 30.2 | 60 | 3.47 | 0.13 | 0.30 | 48.4 |
|  | 220 | 35.0 | 72 | 3.32 | 0.19 | 0.35 | 46.4 |
|  | 315 | 35.8 | 76 | 3.45 | 0.17 | 0.31 | 42.1 |
|  | 360 | 50.7 | 83 | 4.15 | 0.22 | 0.25 | 43.6 |
|  | 156 | 7.5 | 79 | 0.53 | 0.44 | 0.17 | 140.0 |
|  | 291 | 14.8 | 54 | 0.73 | 0.17 | 0.15 | 13.9 |
|  | 186 | 20.5 | 71 | 2.14 | 0.07 | 0.29 | 29.7 |
|  | 205 | 27.1 | 87 | 2.25 | 0.07 | 0.33 | 25.3 |
|  | 254 | 39.1 | 75 | 3.07 | 0.10 | 0.20 | 26.4 |

**Table 1-2 Analysis data of topsoil samples from 146 tea gardens in Guizhou Province(mg·kg^-1^)**

| Region | Mn | Cu | Zn | Mo | Ge | Se | Sr |
| --- | --- | --- | --- | --- | --- | --- | --- |
| Southeast region  （Qiandon-gnan State;n=21） | 142 | 16.6 | 60 | 2.15 | 0.10 | 0.29 | 28.4 |
|  | 260 | 18.6 | 84 | 2.08 | 0.12 | 0.24 | 33.2 |
|  | 231 | 33.4 | 95 | 3.16 | 0.13 | 0.18 | 33.4 |
|  | 515 | 15.0 | 73 | 1.04 | 0.07 | 0.45 | 31.9 |
|  | 417 | 15.5 | 88 | 1.16 | 0.11 | 0.42 | 34.2 |
|  | 542 | 15.3 | 90 | 1.03 | 0.13 | 0.37 | 33.9 |
|  | 181 | 12.3 | 62 | 1.35 | 0.23 | 0.43 | 39.0 |
|  | 215 | 14.1 | 29 | 1.36 | 0.20 | 0.24 | 33.6 |
|  | 360 | 16.1 | 37 | 1.15 | 0.19 | 0.15 | 19.5 |
|  | 472 | 10.5 | 122 | 1.12 | 0.13 | 0.13 | 29.7 |
|  | 247 | 10.7 | 134 | 0.79 | 0.10 | 0.26 | 32.2 |
|  | 247 | 12.4 | 97 | 0.90 | 0.11 | 0.22 | 32.9 |
|  | 582 | 34.6 | 89 | 0.86 | 0.25 | 0.12 | 50.4 |
|  | 410 | 45.9 | 73 | 1.69 | 0.22 | 0.20 | 86.4 |
|  | 357 | 34.2 | 29 | 3.61 | 0.13 | 0.19 | 40.9 |
|  | 460 | 29.8 | 84 | 2.70 | 0.13 | 0.13 | 68.6 |
|  | 335 | 39.6 | 59 | 1.56 | 0.19 | 0.27 | 67.8 |
|  | 360 | 77.7 | 47 | 2.19 | 0.25 | 0.33 | 66.8 |
|  | 505 | 21.9 | 75 | 0.72 | 0.19 | 0.24 | 49.7 |
|  | 480 | 22.8 | 83 | 1.16 | 0.31 | 0.16 | 108.5 |
|  | 536 | 66.6 | 89 | 2.70 | 0.34 | 0.25 | 61.1 |

**Table 1-3 Analysis data of topsoil samples from 146 tea gardens in Guizhou Province(mg·kg^-1^)**

| Region | Mn | Cu | Zn | Mo | Ge | Se | Sr |
| --- | --- | --- | --- | --- | --- | --- | --- |
| South region  （Qiannan State; n=27） | 37 | 4.0 | 27 | 0.99 | 0.14 | 0.25 | 39.6 |
|  | 41 | 4.7 | 40 | 0.99 | 0.16 | 0.39 | 55.0 |
|  | 51 | 3.6 | 38 | 0.75 | 0.22 | 0.46 | 59.9 |
|  | 34 | 4.5 | 25 | 0.88 | 0.16 | 0.35 | 42.1 |
|  | 49 | 5.3 | 53 | 0.91 | 0.17 | 0.52 | 49.4 |
|  | 47 | 6.6 | 34 | 0.65 | 0.18 | 0.72 | 45.3 |
|  | 105 | 9.4 | 62 | 0.50 | 0.47 | 0.43 | 86.4 |
|  | 186 | 10.0 | 66 | 0.52 | 0.31 | 0.33 | 76.1 |
|  | 166 | 10.6 | 71 | 0.57 | 0.46 | 0.41 | 66.5 |
|  | 128 | 13.8 | 50 | 0.96 | 0.23 | 0.72 | 87.4 |
|  | 212 | 15.4 | 56 | 0.85 | 0.26 | 0.55 | 77.3 |
|  | 129 | 16.8 | 49 | 0.83 | 0.35 | 0.57 | 68.6 |
|  | 244 | 45.5 | 101 | 2.04 | 0.10 | 0.60 | 57.9 |
|  | 88 | 50.0 | 49 | 2.25 | 0.11 | 0.83 | 70.8 |
|  | 193 | 44.0 | 62 | 1.51 | 0.07 | 0.57 | 26.5 |
|  | 124 | 46.0 | 93 | 2.38 | 0.09 | 0.44 | 56.8 |
|  | 109 | 65.9 | 128 | 2.53 | 0.07 | 0.32 | 74.2 |
|  | 140 | 81.3 | 152 | 2.16 | 0.09 | 0.28 | 73.2 |
|  | 186 | 28.6 | 75 | 2.25 | 0.08 | 0.36 | 39.5 |
|  | 207 | 32.2 | 81 | 1.41 | 0.16 | 0.55 | 44.4 |
|  | 140 | 31.2 | 77 | 1.47 | 0.12 | 0.49 | 44.8 |
|  | 242 | 27.5 | 46 | 1.95 | 0.06 | 0.70 | 47.8 |
|  | 203 | 41.8 | 54 | 1.50 | 0.08 | 0.38 | 52.5 |
|  | 281 | 37.1 | 62 | 1.42 | 0.07 | 0.45 | 52.8 |
|  | 315 | 67.0 | 92 | 2.97 | 0.15 | 0.72 | 57.5 |
|  | 324 | 47.5 | 153 | 2.75 | 0.13 | 0.85 | 58.1 |
|  | 290 | 22.0 | 92 | 2.59 | 0.09 | 0.52 | 60.0 |

**Table 1-4 Analysis data of topsoil samples from 146 tea gardens in Guizhou Province(mg·kg^-1^)**

| Region | Mn | Cu | Zn | Mo | Ge | Se | Sr |
| --- | --- | --- | --- | --- | --- | --- | --- |
| Central region  （Guiyang City；Anshun City; n=22） | 271 | 69.0 | 59 | 4.94 | 0.07 | 1.43 | 89.0 |
|  | 230 | 40.7 | 61 | 2.53 | 0.08 | 0.94 | 70.9 |
|  | 378 | 84.6 | 143 | 4.31 | 0.14 | 1.32 | 65.4 |
|  | 296 | 72.8 | 54 | 4.23 | 0.09 | 1.19 | 89.5 |
|  | 386 | 94.7 | 117 | 5.02 | 0.07 | 0.92 | 80.7 |
|  | 582 | 85.0 | 130 | 4.58 | 0.09 | 0.87 | 78.2 |
|  | 650 | 11.4 | 74 | 1.12 | 0.13 | 0.45 | 39.7 |
|  | 254 | 20.1 | 63 | 3.25 | 0.18 | 0.37 | 26.2 |
|  | 471 | 23.0 | 75 | 2.36 | 0.12 | 0.52 | 26.0 |
|  | 88 | 17.1 | 42 | 1.12 | 0.09 | 0.30 | 38.5 |
|  | 100 | 15.9 | 69 | 1.83 | 0.08 | 0.28 | 153.5 |
|  | 210 | 21.5 | 81 | 1.75 | 0.13 | 0.35 | 77.2 |
|  | 840 | 72.0 | 213 | 2.16 | 0.29 | 0.86 | 52.0 |
|  | 764 | 76.5 | 212 | 2.04 | 0.28 | 0.82 | 52.8 |
|  | 918 | 87.0 | 209 | 1.80 | 0.30 | 0.61 | 56.5 |
|  | 563 | 103.5 | 153 | 1.80 | 0.24 | 0.74 | 46.2 |
|  | 510 | 92.0 | 181 | 1.32 | 0.32 | 0.50 | 42.6 |
|  | 659 | 107.0 | 181 | 1.23 | 0.34 | 0.55 | 43.4 |
|  | 750 | 90.5 | 117 | 2.57 | 0.25 | 0.77 | 88.0 |
|  | 623 | 60.0 | 182 | 2.63 | 0.38 | 0.79 | 101.0 |
|  | 765 | 68.0 | 180 | 2.23 | 0.37 | 0.64 | 88.3 |
|  | 980 | 56.5 | 177 | 2.31 | 0.26 | 0.34 | 79.5 |

**Table 1-5 Analysis data of topsoil samples from 146 tea gardens in Guizhou Province(mg·kg^-1^)**

| Region | Mn | Cu | Zn | Mo | Ge | Se | Sr |
| --- | --- | --- | --- | --- | --- | --- | --- |
| Northwest region  （Zunyi City; n=29） | 738 | 25.1 | 68 | 0.71 | 0.17 | 0.52 | 44.5 |
|  | 358 | 22.0 | 77 | 0.57 | 0.18 | 0.39 | 37.2 |
|  | 362 | 19.7 | 82 | 0.52 | 0.19 | 0.35 | 46.5 |
|  | 798 | 23.9 | 124 | 0.72 | 0.28 | 0.47 | 40.6 |
|  | 917 | 28.0 | 110 | 0.64 | 0.38 | 0.58 | 44.2 |
|  | 932 | 28.8 | 113 | 0.60 | 0.36 | 0.50 | 50.9 |
|  | 909 | 84.9 | 145 | 3.15 | 0.36 | 1.49 | 34.7 |
|  | 504 | 86.5 | 181 | 1.81 | 0.34 | 1.17 | 28.5 |
|  | 845 | 106.0 | 144 | 1.22 | 0.32 | 1.20 | 26.0 |
|  | 703 | 111.0 | 138 | 1.34 | 0.34 | 1.34 | 29.1 |
|  | 620 | 91.5 | 165 | 1.01 | 0.34 | 1.46 | 25.5 |
|  | 765 | 113.6 | 172 | 1.27 | 0.29 | 0.67 | 29.8 |
|  | 877 | 28.8 | 101 | 1.93 | 0.16 | 0.58 | 48.6 |
|  | 588 | 30.4 | 107 | 1.89 | 0.18 | 0.52 | 50.1 |
|  | 571 | 29.0 | 103 | 1.84 | 0.17 | 0.47 | 49.6 |
|  | 776 | 19.2 | 87 | 1.39 | 0.17 | 0.58 | 29.1 |
|  | 344 | 16.7 | 75 | 1.19 | 0.13 | 0.48 | 26.2 |
|  | 221 | 24.4 | 93 | 1.61 | 0.13 | 0.39 | 24.1 |
|  | 182 | 17.3 | 80 | 1.50 | 0.15 | 0.35 | 24.8 |
|  | 759 | 28.7 | 183 | 2.27 | 0.16 | 0.44 | 22.6 |
|  | 695 | 39.5 | 225 | 3.68 | 0.19 | 1.62 | 22.3 |
|  | 823 | 21.5 | 111 | 2.32 | 0.15 | 0.77 | 27.2 |
|  | 638 | 39.2 | 219 | 4.37 | 0.18 | 0.65 | 20.3 |
|  | 539 | 40.7 | 216 | 3.80 | 0.16 | 0.49 | 21.1 |
|  | 817 | 39.7 | 179 | 3.97 | 0.16 | 0.55 | 52.0 |
|  | 876 | 69.1 | 251 | 2.55 | 0.15 | 0.58 | 26.7 |
|  | 761 | 77.5 | 208 | 2.30 | 0.14 | 0.42 | 26.5 |
|  | 797 | 117.2 | 119 | 3.18 | 0.33 | 1.17 | 98.8 |
|  | 662 | 115.0 | 117 | 2.31 | 0.35 | 1.25 | 103.5 |

**Table 1-6 Analysis data of topsoil samples from 146 tea gardens in Guizhou Province(mg·kg^-1^)**

| Region | Mn | Cu | Zn | Mo | Ge | Se | Sr |
| --- | --- | --- | --- | --- | --- | --- | --- |
| Southwest region  （Qianxinan State; n=20） | 532 | 86.7 | 102 | 3.98 | 0.33 | 1.13 | 103.5 |
|  | 615 | 91.9 | 93 | 4.18 | 0.29 | 0.98 | 118.5 |
|  | 447 | 109.0 | 103 | 3.83 | 0.29 | 0.75 | 125.5 |
|  | 536 | 91.1 | 66 | 2.10 | 0.24 | 0.82 | 103.0 |
|  | 403 | 117.2 | 71 | 4.81 | 0.24 | 0.90 | 119.0 |
|  | 394 | 98.5 | 76 | 4.92 | 0.24 | 0.74 | 123.5 |
|  | 594 | 96.8 | 62 | 5.04 | 0.32 | 1.25 | 153.5 |
|  | 485 | 60.7 | 75 | 4.25 | 0.45 | 1.07 | 178.3 |
|  | 472 | 109.5 | 86 | 4.87 | 0.46 | 1.19 | 164.5 |
|  | 687 | 25.4 | 49 | 1.11 | 0.06 | 0.47 | 31.8 |
|  | 479 | 21.6 | 63 | 1.04 | 0.07 | 0.38 | 35.5 |
|  | 532 | 17.4 | 55 | 0.96 | 0.11 | 0.57 | 35.2 |
|  | 695 | 14.8 | 52 | 1.10 | 0.17 | 0.43 | 30.7 |
|  | 787 | 15.5 | 46 | 1.20 | 0.10 | 0.38 | 36.0 |
|  | 694 | 18.0 | 65 | 1.07 | 0.19 | 0.44 | 37.8 |
|  | 561 | 70.5 | 171 | 3.78 | 0.21 | 0.87 | 67.6 |
|  | 514 | 95.4 | 209 | 4.05 | 0.20 | 0.39 | 59.1 |
|  | 409 | 75.2 | 186 | 3.41 | 0.25 | 0.92 | 70.9 |
|  | 379 | 72.5 | 174 | 3.81 | 0.22 | 0.74 | 68.8 |
|  | 226 | 62.7 | 158 | 2.97 | 0.26 | 0.45 | 74.8 |
